# High-Resolution Genotyping via Whole Genome Hybridizations to Microarrays Containing Long Oligonucleotide Probes

**DOI:** 10.1371/journal.pone.0014178

**Published:** 2010-12-02

**Authors:** Yan Fu, Nathan M. Springer, Kai Ying, Cheng-Ting Yeh, A. Leonardo Iniguez, Todd Richmond, Wei Wu, Brad Barbazuk, Dan Nettleton, Jeff Jeddeloh, Patrick S. Schnable

**Affiliations:** 1 Department of Agronomy, Iowa State University, Ames, Iowa, United States of America; 2 Department of Plant Biology, University of Minnesota, St Paul, Minnesota, United States of America; 3 Interdepartmental Genetics Graduate Program, Iowa State University, Ames, Iowa, United States of America; 4 Department of Genetics, Development and Cell Biology, Iowa State University, Ames, Iowa, United States of America; 5 Center for Plant Genomics, Iowa State University, Ames, Iowa, United States of America; 6 Roche NimbleGen, Inc., Madison, Wisconsin, United States of America; 7 Department of Biology and the Genetics Institute, University of Florida, Gainesville, Florida, United States of America; 8 Department of Statistics, Iowa State University, Ames, Iowa, United States of America; United States Department of Agriculture, Agricultural Research Service, United States of America

## Abstract

To date, microarray-based genotyping of large, complex plant genomes has been complicated by the need to perform genome complexity reduction to obtain sufficiently strong hybridization signals. Genome complexity reduction techniques are, however, tedious and can introduce unwanted variables into genotyping assays. Here, we report a microarray-based genotyping technology for complex genomes (such as the 2.3 GB maize genome) that does not require genome complexity reduction prior to hybridization. Approximately 200,000 long oligonucleotide probes were identified as being polymorphic between the inbred parents of a mapping population and used to genotype two recombinant inbred lines. While multiple hybridization replicates provided ∼97% accuracy, even a single replicate provided ∼95% accuracy. Genotyping accuracy was further increased to >99% by utilizing information from adjacent probes. This microarray-based method provides a simple, high-density genotyping approach for large, complex genomes.

## Introduction

The ability to rapidly determine genotypes at many loci in numerous individuals is critical to furthering our understanding of the inheritance of complex traits and for developing improved strategies for plant breeding. The use of molecular markers based on isozymes, RFLPs (restriction fragment length polymorphisms), SSRs (simple sequence repeats) and CAPS (cleaved amplified polymorphic sequences) genetic markers allowed for the construction of early genetic maps. However, these initial genotyping technologies were of relatively low throughput and required significant effort per data point.

A number of technologies have been developed for high-throughput genotyping (reviewed by [1], [2], [3], [4]). There are additional approaches that combine the use of microarrays and restriction digests such as diversity array technology (DArT) [5] and restriction site associated DNA (RAD) tags [6] to assay up to several thousand markers. These high-throughput approaches vary substantially in number of markers, amount of information required for development, accuracy, ease of application and data analysis. In particular, there are limitations on the application of some of these methods to species with large, complex genomes.

The wide-spread availabilities of genomic and EST sequences in many species have led to the development of markers based on SNPs (single nucleotide polymorphisms). Several companies have developed high-throughput technologies that can genotype up to several hundred thousand SNPs in a single reaction [7], [8]. Flibotte *et al.* [9] have reported the detection of SNPs in *C. elegans* (genome size 0.1 GB) using whole genome hybridization to arrays containing oligo probes designed based on the sequences of known SNPs (SNP-CGH). SNPs can be extremely valuable molecular markers but effort is required for the discovery and validation of SNPs, as well as for assay development. Alternative genotyping approaches that do not require prior knowledge of SNPs have also been developed. For example, RAD tags can be sequenced to discover and map SNPs [10]. Alternatively, SNPs and SFPs (single feature polymorphisms) can be detected by hybridizing genomic DNA or RNA to short oligonucleotide microarrays that contain short (∼25 mer) oligonucleotides [11], [12], [13], [14], [15], [16], [17]. Longer oligonucleotide probes have also been used to detect SFPs caused by indel (insertion/deletion) polymorphisms [18], [19], [20]. Although this process is quite efficient in organisms with relatively small genomes, it has proven less successful for detecting polymorphisms in genomic DNA from organisms with larger genomes, such as maize (2.3 GB, [21]), due to a lack of sufficient signal strength. To obtain sufficient signal strengths in such species it is necessary to utilize RNA or reduced complexity (e.g., high C_o_ *t* or methylation filteration) DNA. Unfortunately, these approaches introduce variables, such as expression level or filtration efficiency, that can complicate genotyping efforts.

Previously we developed a custom long oligonucleotide microarray that yielded strong signals from whole genome hybridizations. This array was used to assess structural variation between the two maize inbreds B73 and Mo17 [22]. In that study ∼200,000 probes were identified that exhibited highly differential and discriminatory hybridization signals between B73 and Mo17 genomic DNA. These hybridization differences were typically caused by the presence of multiple SNPs, small IDPs (InDel Polymorphisms), and CNVs (copy number variants), including PAVs (presence absence variants), rather than by single SNPs [22]. To demonstrate the utility using these polymorphic probes as molecular markers for whole-genome genotyping in a large, complex genome, in this study two recombinant inbred lines (RILs) from the maize IBM mapping population [23] were randomly selected and analyzed via array comparative genomic hybridization (aCGH). In this report, we demonstrate the utility of using long oligonucleotide microarrays for high-throughput mapping in maize without the need to apply complexity reduction methods. The attractive features of this system are first, that it does not require prior knowledge of polymorphisms; second, that the genotyping results are highly accurate; third, high probe density allows for the fine-mapping of recombination breakpoints; and fourth, data analysis can be relatively simple.

## Results

### Identification of a large number of CGH-based polymorphisms

The first objective was to identify polymorphic probes that could be scored in the RILs. To identify such probes we used data derived from hybridization of the two parental genotypes (B73 and Mo17) to the microarray (all microarray data were deposited into GEO under Series# GSE16938). The microarray platform contained 1,262,421 probes that could each be unambiguously mapped to a single location (i.e., uniquely mapped) in the maize genome and that we therefore concluded are non-repetitive [22]. Following normalization and linear modeling (see [22] for full details), we identified 225,867 probes that exhibited significant differences in hybridization between B73 and Mo17 genomic DNA at a false discovery rate (FDR) cut-off of <0.0001 (Table 1). The vast majority (91%) of these probes have higher hybridization signal intensities in B73 than in Mo17 and are referred to as B>M probes. Because the microarray was designed based on the reference B73 genomic sequence this observation is an expected consequence of ascertainment bias. B>M probes may have either of three possible characteristics. They can occur due to the existence of polymorphisms within the probe sequence between the two genotypes, due to the presence of more copies of the probe sequence in B73 than in Mo17, or due to a deletion of the probe sequence from the Mo17 genome. Because all probes were designed based on the B73 reference genome sequence, those probes that exhibit M>B hybridization ratios are expected to be present at higher copy number in the Mo17 genome than in the B73 genome.

**Table 1:** Comparison of genotyping using different subsets of probes and multiple analysis methods.

	B>M (all^a^)		B>M (2-FC^b^)		M>B (all^a^)		M>B (2-FC^b^)	
Number probes	204934		164728		20933		8394	
RIL - M0022	% Calls^c^	% Consistent^d^	% Calls^c^	% Consistent^d^	% Calls^c^	% Consistent^d^	% Calls^c^	% Consistent^d^
Linear model calls (Method I)								
	95.2%	94.9%	95.3%	96.7%	85.9%	82.8%	78.4%	83.4%
Simple model calls (Method II)								
Replicate 1	93.1%	91.9%	93.4%	94.5%	74.2%	76.6%	73.6%	80.6%
Replicate 2	93.8%	93.6%	94.8%	96.0%	74.6%	79.5%	76.0%	81.8%
Consistency among replicates								
Both same call	84.0%	95.3%	87.3%	96.7%	44.2%	81.9%	49.9%	85.4%
Opposing calls^5^	4.2%		2.0%		11.5%		7.4%	
RIL -M0023	% Calls^c^	% Validated^d^	% Calls^c^	% Validated^d^	% Calls^c^	% Validated^d^	% Calls^c^	% Validated^d^
Linear model calls (Method I)								
	92.5%	96.7%	93.3%	97.1%	80.9%	86.4%	74.2%	87.7%
Simple model calls (Method II)								
Replicate 1	93.7%	93.9%	94.8%	96.3%	71.2%	80.5%	72.4%	84.7%
Replicate 2	96.6%	94.4%	97.4%	96.5%	73.2%	81.5%	72.7%	83.8%
Replicate 3	95.2%	94.2%	96.5%	96.5%	77.3%	82.9%	78.9%	85.4%
Consistency among replicates								
Same call in all replicates	90.9%	95.4%	94.1%	96.9%	58.0%	83.9%	60.9%	86.0%
Opposing calls e	1.8%		0.6%		3.3%		2.1%	

a The B>M or M>B (all) refers to all probes with a FDR<0.05 in a comparison of B73 and Mo17.

b The B>M or M>B (2-FC) probes refers to the subset of polymorphic probes that have a FDR<0.0001 and a minimum of 2-fold change between B73 and Mo17.

c The % calls is the percent of SFPs that could be assigned a genotype using an analysis method.

d The % consistent refers to the percentage of polymorphic probes that were assigned the same genotype in previously generated genotyping data.

e The opposing calls are those for which the same probe was assigned different genotypes in different replicates.

An additional filter was applied to the derived probe list to cull for only the most utilitarian probes. This additional filter identified 173,122 probes that exhibited a minimum fold change of 2 (Table 1). This filter removed ∼20% of the B>M probes and nearly 60% of the M>B probes.

The remaining 173,122 probes were annotated based on their genomic map positions relative to the B73 reference genome [21] and conservation in the Mo17 genome. Each probe sequence was compared with an ∼5X whole-genome shotgun (WGS) sequence of Mo17 generated by the Joint Genome Institute using 454 sequencing technology (pre-publication access to these sequences was kindly provided by Dan Rokshar). Each probe was assigned a value of perfect match (100% identity and coverage), conserved (>90% coverage and identity) or “no match” (<90% coverage and/or identity). The majority (59%) of the B>M probes had no match in the collection of Mo17 WGS sequence reads, while only 1% had a perfect match (Table S1). Hence, as expected most (99%) of the B>M probe sequences are either absent from Mo17 or are polymorphic relative to B73. In contrast, the majority (51%) of the M>B probes had a perfect match in the collection of Mo17 WGS sequence reads, while only 13% did not have a match. Collectively, this set of polymorphic probes consisted of 173,122 probes, which included at least 12,000 probes for each of the 10 maize chromosomes (Table S2).

### Assessment of data analysis and subsets of polymorphic probes

**Figure 1:**
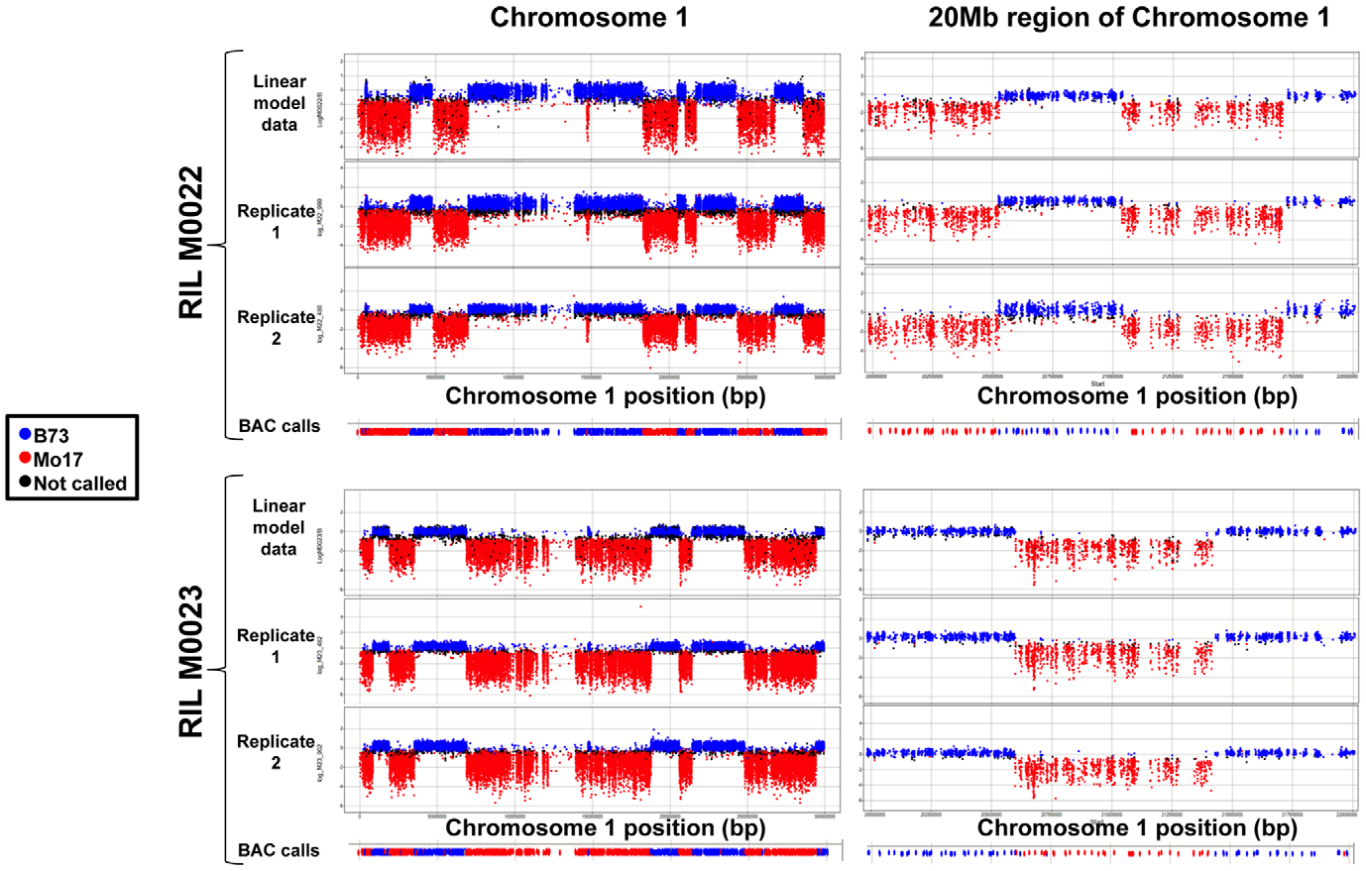
Comparisons of analytical approaches for CGH-based mapping. Data for B>M probes located on chromosome 1 that exhibit at least a 2-fold change (n = 26,953) were plotted following the use of different data analysis approaches. The upper set of plots display data for the RIL M0022 while the lower panels show data for the RIL M0023. The plots on the left and right display probes from the entire chromosome 1 and a close-up view of a 20 Mb region of chromosome 1 (positions 200 Mb–220 MB), respectively. For each set of plots the first panel provides visualization genotyping calls and log2 (RIL/B73) ratios following normalization and analysis using a linear model of multiple replications (Method I). The second and third panels show the genotyping calls and log_2_(RIL/B73) ratios based on the analysis of a single replicate of data that was normalized using standard NimbleScan approaches (Method II). The final plot shows the genotyping calls for each BAC (n = 1,369) using data from replicate 1 (Method III).

The potential of these polymorphic probes for genetic mapping was evaluated using two B73xMo17 recombinant inbred lines (IBM RILs; [23]) both of which we and others had previously genotyped using ∼10,000 markers [24], [25]. To enhance the utility of microarray-based genotyping we assessed the importance of hybridization replication, compared various methodologies for data analysis, and determined the effects of polymorphism types upon the accuracy of genotype determinations. Comparisons of the number of markers and accuracy of several different analytical approaches, including linear modeling of replicates, simple assessment of relative signals from a single replicate, and BAC-based genotyping were considered most germane. A visualization of the results obtained for chromosome 1 mapping was made for each of these approaches and is depicted in Figure 1.

The first analytical approach (Method I) involved the use of normalization methodology and subsequent estimation of the errors accounted for by dye and genotype effects upon the signal as determined via a linear model. This approach allowed for statistical contrasting of RIL vs. B73 and RIL vs. Mo17 at each probe using two hybridizations (See Methods for details). q-values were obtained for each of these contrasting comparisons and each probe was assigned to one of four classes in each RIL. Probes that were significantly different (q<0.05) from B73 but not from Mo17 in the RIL hybridizations were assigned a genotype of B (Class I) and probes that were different from Mo17 but not from B73 were assigned a genotype of M (Class II). Some probes exhibited significant differences as compared to both parental lines (Class III) or were not significant in either of the two comparisons (Class IV). These later two classes may reflect non-polymorphic probes, residual heterozygosity or complex genome arrangements of gene families. Based on the broad genomic distribution of these probes (black dots in Figure 1) it is unlikely that residual heterozygosity is a major cause. Method I was able to assign genotypes for 93–95% of B>M probes in both RILs and was unaffected by the use of filtering based on a fold change (Table 1). However, substantially fewer of the M>B probes (74–86%) could be assigned genotypes (Table 1). Previously obtained genotyping results (from [25]) were used to validate the array-based genotyping calls (see Methods for details). As expected, consistency rates were substantially higher for the B>M probes than for the M>B probes and the use of the filtered probe set provided only a slight improvement in the validation (consistency) rate. While Method I provides robust results, it requires substantial bioinformatics expertise, as well as replication of hybridizations, factors that could discourage the broad adoption of this microarray-based genotyping platform.

Therefore, a more streamlined analytic method (Method II) was considered. This method employed a single hybridization and thus vastly reduced the complexity of the required computational analyses. In Method II, spatially normalized data from a single array were analyzed and hybridization contrasts were considered without applying statistical methods (See Methods for details). Our goal was to assess the relative loss of accuracy and information achieved using this relatively simple method of analysis and a single hybridization as compared to the robust Method I. The genotype for each probe was assigned by calculating the hybridization difference of the RIL and B73 relative to Mo17 and B73 [(RIL-B73)/(Mo17-B73)]. Probes with values near zero have hybridization intensities that are more similar to B73 than to Mo17, while values near 1 have hybridization intensities more similar to Mo17 than to B73. All probes with values less than 0.33 were assigned a genotype of B73 and probes with values greater than 0.66 were assigned a genotype of Mo17. The remaining probes were not classified (visualization provided in Figure 1). This approach assigned genotypes to slightly fewer probes than did the linear model (Method I) and had a slightly lower validation rate. Even so, this less complex analytic method still provided genotyping calls for >90% of the B>M probes and these calls were ∼95% consistent with independently determined genotypes. Note that the filtered set of B>M probes provided substantially more benefit for Method II than for Method I. Consequently, rigorous filtering of probes is more critical when using a single hybridization (Method II) than when data from multiple hybridizations (Method I) are available. Based on a comparison of genotyping calls between two replicates (only two of the M0023 Cy3 replicates was used to enhance the consistency of analyses) the majority of the genotype assignments determined using B>M probes were consistent between pairs of replicates and only 2–4% of probes were called as different genotypes in the two replicates. As expected, the performance for the M>B probes was substantially lower for this approach. Only 40–60% of the M>B probes were consistently assigned to the same genotype across independent replicates and the rate of inconsistent calls between the replicates was higher (Table 1).

**Figure 2:**
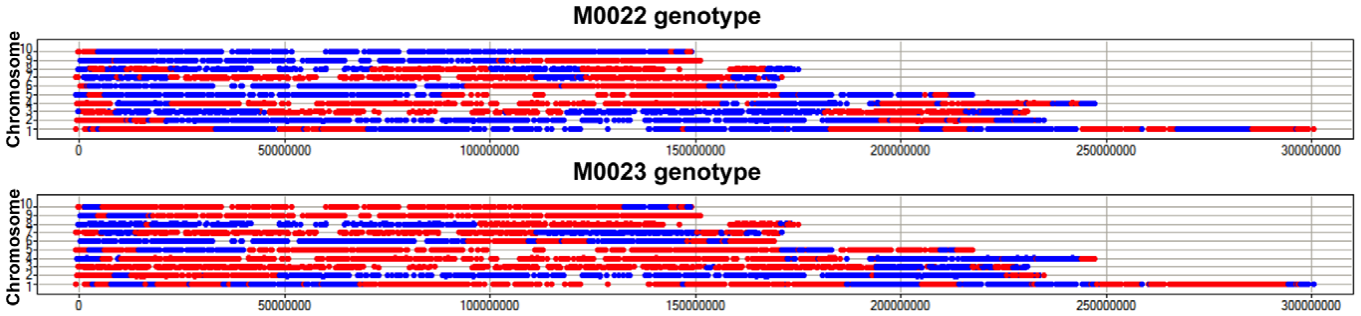
Visualization of whole genome genotypes of two RILs. The genotype of each BAC with at least 5 filtered B>M probes (n = 7,978 that were called in both lines) was determined and color coded (B73 – blue; Mo17 – red). BACs were then plotted according to their physical positions along a chromosome (x-axis) and by chromosome (y-axis). This visualization was created using a single replicate of data.

Next, we investigated the utility of assigning genotypes to RILs based on a series of probes that were closely linked and that exhibited similar genotyping calls. The physical map of the maize genome is quite accurate at a resolution of single BACs [21]. However, the order and orientation of DNA sequences within BACs is often not known. This can lead to incorrect fine-scale arrangements in the order of probes in our genotyping data. Assigning each BAC a genotype in each RIL alleviates this problem. BAC-level genotyping is also expected to increase the accuracy of genotyping assignments because it allows for a genotyping assignment to be made using multiple probes located on the same BAC. In addition, doing so simplifies data visualization by reducing the number of data points. For this analysis we used only those B>M probes that had a minimum of a 2-fold change because this set exhibited the greatest accuracy in both Methods I and II. To be assigned a genotype a BAC had to have at least 5 probes that were assigned a genotype of B73 or Mo17 and these probes had to exhibit at least 80% genotype agreement within the BAC. Using this approach, genotypes could be assigned to over 95% the 8,497 BACs that contain at least 5 polymorphic probes (Method III, Table 2). The different methods of analysis were able to assign a genotype for slightly different sets of BACs (Table 2) but for BACs that were assigned genotypes with both methods there was 100% agreement of the genotyping calls made by the different approaches. By comparing the genotyping assignments with the genotyping data of Liu *et al.* [25] we could demonstrate >99% accuracy for each of these approaches. This approach of assigning a genotype for each BAC in each RIL allows for simple visualization of the genotyping calls (Figures 1 and 2).

**Table 2:** Genotyping calls by BAC (Method III).

	# BACs assigned genotype	% Consistent with PCR genotyping
M0022		
Linear model	8333	99.19%
Simple model		
Replicate 1	8206	99.18%
Replicate 2	8264	99.22%
Consensus^a^	8036	99.23%
M0023		
Linear model	8262	99.44%
Simple model		
Replicate 1	8289	99.47%
Replicate 2	8351	99.51%
Replicate 3	8325	99.47%
Consensus	8273	99.47%

a The consensus line specifies the number of BACs that were assigned the same genotype in all replicates.

**Figure 3:**
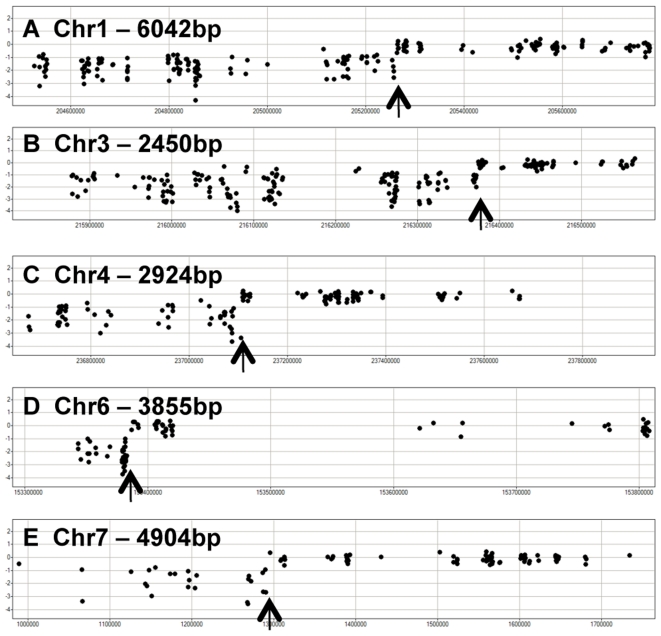
High-resolution of recombination break-points. Several plots show detailed views of the CGH mapping data near recombination events in the RIL M0022. The log2(M0022/B73) value is plotted along the y-axis for each of the B>M probes (q<0.0001 and fold-change>2) in five genomic regions. The arrowheads indicate the position of the recombination event and the label indicates the chromosome and the base pair resolution of the recombination event.

We assessed the resolution of the genotyping data that was generated by CGH. While some recombination break-points can only be resolved within ∼100 kb due to lack of polymorphic probes in the region of the recombination event other cross-overs can be resolved at quite high resolution. Figure 3 provides five examples of highly resolved cross-overs in the M0022 RIL. The exact location of these five cross-overs could be identified within 2,450 to 6,042 base pairs. The ability to pinpoint the location of recombination events was influenced by the number of polymorphic probes within the region.

## Discussion

This report documents the feasibility of genotyping complex genomes via a microarray-based method that does not require the use of methods to reduce genome complexity prior to hybridization. To do so, we used long oligonucleotides, which increased our ability to detect signal from genomic DNA and leveraged the abundance of frequent and widely distributed differences in DNA sequences between haplotypes as molecular markers. This approach is particularly valuable because it allows for mapping experiments even in the absence of any prior knowledge of polymorphisms between the parents of a mapping population, does not require extensive laboratory manipulation and can be performed using a single hybridization replicate. Additional experiments (data not shown) have demonstrated that this approach can be used to genotype individuals containing heterozygosity (such as F2 individuals) as well as homozygous RILs.

### Comparison of linear model (Method I) and single array (Method II) analyses

Careful comparisons enabled us to determine the numbers of markers that could be genotyped, and their validation rates using replicated data analyzed using a linear model (Method I) as well as a more simplified analysis (Method II) conducted on non-replicated data. Replicates did provide slightly high numbers of markers that could be scored and yielded genotyping data that was validated at slightly higher rates. However, when probes were filtered to use only those that exhibited at least 2-fold change between the parental lines, the overall validation rates for Methods I and II were quite similar. We found that assigning genotypes to each BAC based on the occurrence of multiple polymorphic probes within the same BAC provided highly accurate genotyping scores. Indeed, this method resulted in nearly perfect validation rates (>99%). If it is desired to perform fine-scale mapping of recombination break-points to the highest possible resolution it would be possible to first conduct a BAC-based analysis to map recombination breakpoints to a BAC-level resolution. Subsequently, the analysis of individual probes within those BACs near the recombination breakpoint could further define the position of the recombination breakpoint.

### Genotyping accuracy of different classes of probes

Because this method relies on long oligonucleotide probes many of the detected polymorphisms are likely to be structural variants. Because our probes were designed based upon the sequence of the B73 reference genome [21], each exhibits a perfect match to B73. Therefore, all probes with higher hybridization intensities in Mo17 than in B73 (M>B probes) are expected to represent sequences that are present in more copies in Mo17 than in B73 (CNVs). In contrast, the sequences detected by probes having higher hybridization intensities in B73 than in Mo17 (B>M probes) are likely to: 1) exhibit multiple sequence differences (SNPs and/or IDPs) between B73 and Mo17; 2) be absent from the Mo17 genome (PAVs); or 3) exist in higher copy number in B73 than in Mo17 (CNV). Because only probes that had a single match to the B73 reference genome were used in this analysis, it is likely that most of the B>M probes are from the first two classes. Consistent with this view, a comparison of the B>M probes with a collection of Mo17 WGS sequences (∼5X coverage) revealed that many do not have a 90% identical sequence. Although all probes used in this analysis are single copy in the B73 reference genome, it is, however, conceivable that some of the B>M probes match duplicated regions of the actual B73 genome that were either not sequenced or that were inadvertently collapsed into single copies during genome assembly. Hence, a small fraction of the B>M probe sequences may in fact exist at higher copy numbers in the B73 genome than in the Mo17 genome. Consistent with this possibility, a small fraction (1%) of the B>M probes had a perfect match to Mo17. This result is unexpected if indeed these probe sequences exist as single copy sequences in the B73 genome. The 13% of the M>B probes that did not have matches in the collection of Mo17 WGS sequence reads could be the result of inadequate sampling of the Mo17 genome. Although a small fraction of probes exhibit hybridization patterns that differ from expectations for various reasons, the overall mapping accuracy and resolution generated using this technology is high.

It is noteworthy that the proportion of M>B probes that could be called and their validation rates were much lower than for the B>M probes. This likely reflects the fact that copy number variants (CNVs) could be in either the *cis* or *trans* configuration. If the multiple copies of a probe sequence are not closely linked in the Mo17 genome (i.e., in a *trans* configuration) they would be expected to segregate among RILs, potentially yielding novel hybridization signals (i.e., not similar to either of the parental signals).

### Mapping strategy

In the experiments reported here we used an array that contained ∼2.1 M probes. Roche NimbleGen also offers a customizable 12-plex suite of arrays. It contains 12 sets of the same 135,000 probes on a standard glass slide. Using the data obtained from a single dye-channel such a 12-plex array can be used to genotype 24 lines; these arrays have significant advantages from the perspectives of cost and efficiency. We have considered how best to modify our mapping strategy to accommodate the fact that each genotype will be analyzed with fewer probes (135,000 vs. 2.1 M). As a consequence of the high degree of sequence polymorphism in maize ∼10% of the probes on our 2.1 M array (i.e., ∼200,000) proved to be polymorphic between B73 and Mo17 even though our custom array was designed based on the B73 haplotype without reference to the sequence of the Mo17 haplotype. We expect quantitatively similar results would be obtained when comparing B73 to any other inbred that is not closely related to B73. Indeed, this was observed in comparisons of the inbreds Hp301 and Tx303 to B73 (unpublished observation). But importantly for the design of a mapping strategy, the same probes are not likely not be polymorphic in all comparisons or mapping populations.

We therefore recommend a two-step mapping strategy. In the first step a survey array containing ∼2.1 M probes sampled from the low-copy, genic regions of the genome of interest will be used to identify probes that are informative in a given population via hybridizations to the parents of the mapping population. In the second step, based on the results of these hybridizations, ∼135,000 of the most informative polymorphic probes would be selected and used to construct 12-plex arrays for genotyping members of a mapping population.

We recommend for routine genotyping experiments using those probes that exhibit higher hybridization intensities from the reference genome (e.g., B>M probes) for initial mapping applications because they have higher validation rates. Subsequent analyses could use probes having higher hybridization intensities in the non-reference genome to estimate the relative rates of tandem and dispersed duplications.

## Materials and Methods

### Plant materials

Genomic DNA was isolated from two-week-old seedlings of the inbreds B73 and Mo17 as well as from two IBM RILs: M0022 and M0023. According to previous genotyping results [24] the genomes of these two RILs are ∼56% identical. 1 mg of DNA was labeled using either 5′ Cy3 or Cy5-labeled Random Nonamers (TriLink Biotechnologies). DNA was incubated for 2 hours at 37°C with 100 units (exo-) Klenow fragment (NEB) and dNTP mix (6 mM each in TE; Invitrogen). Labeled samples were then precipitated with NaCl and isopropanol and rehydrated in 25 µl of VWR H20. 34 µg of test and reference samples were combined in a 1.5 ml tube and dried down using a SpeedVac. Samples were resuspended in 12.3 µl of H20 and 31.7 µl of NimbleGen Hybridization Buffer (Roche NimbleGen Inc.) and incubated at 95°C. The combined and resuspended samples were then hybridized to the array for 60–72 hours at 42°C degrees with mixing. Arrays were washed using NimbleGen Wash Buffer System and dried using a NimbleGen Microarray Dryer (Roche NimbleGen, Inc). Arrays were scanned at 5 µm resolution using a GenePix4000B scanner (Axon Instruments). Data were extracted from scanned images using NimbleScan 2.4 extraction software (Roche NimbleGen, Inc.), which allows for automated grid alignment, extraction and generation of data files. For this experiment, five hybridizations were performed and the samples hybridized to each array are as follows: Array 1 M0023 (Cy3)/B73 (Cy5); Array 2 M0022 (Cy3)/B73 (Cy5); Array 3 Mo17 (Cy3)/M0023 (Cy5); Array 4 Mo17 (Cy3)/M0022 (Cy5); Array 5 M0023 (Cy3)/B73 (Cy5).

### CGH data analyses

The probes were mapped to the B73 RefGen_v1 genome sequence [21] with 100% identity and coverage [22] and only probes with a single perfect match were used for this analysis. The integrated genetic and physical map of maize [25] was used to determine the physical location of each genetic marker on B73 RefGen_v1. The hybridization intensity of each mapped probe was estimated within each genotype using LIMMA [26] according to [22]. When applying q<0.0001 cutoff [27], a total of 225,867 probes that exhibited significantly different hybridization signals between B73 and Mo17 were deemed to be polymorphic. This set was further divided and filtered based on which genotype exhibited a higher signal and whether there was at least a 2-fold change in signal intensity between B73 and Mo17 (Table 1).

### CGH-based Genotyping

#### Linear model

A linear model was used to calculate a q-value to estimate the false-discovery corrected probability that a particular probe was different from B73 or from Mo17. For each RIL, a probe was assigned a value of “B73” if it was significantly different from Mo17 (q<0.05) but not from B73 and was assigned a value of “Mo17” if it was significantly different from B73 (q<0.05) but not from Mo17. The probes that were significantly different from both or neither parental lines were not assigned a genotype.

#### Single array based model

A simple model was employed to assign genotype calls using a single replicate of data. The spatially normalized data extracted for each array using the NimbleScan software were imported into Excel. For each probe the value of [(RIL-B73)/(Mo17-B73)] was calculated. Cut-off values of 0.33 and 0.66 were arbitrarily selected for the purpose of this analysis. All probes having values of less than 0.33 was assigned a genotype of B73, while probes having values greater than 0.66 were assigned a genotype of Mo17. Probes with values between 0.33 and 0.66 were not classified. The values for different replicates were subsequently compared to determine the number of genotype assignments that were shared or conflicting for each of the hybridizations.

#### BAC based genotyping

Genotypes of each BAC were determined by comparing the calls for all polymorphic probes within a BAC. Genotypes were assigned to BACs have at least five polymorphic probes and only BACs with at least 80% agreement for the genotypes of all probes within the BAC that were classified as B73 or Mo17. The “consensus” genotyping assignments were assigned when a BAC was assigned the same genotype for each of the replicates for a RIL.

### Validation of genotype assignments

The genotype scores for each of the two RILs were collected from a total of 10,143 markers [25] including IDP markers [24], TIDP markers [28], SNP markers [29] and other markers downloaded from MaizeGDB (http://www.maizegdb.org). If one of these markers was located within 5,000 bp of a probe, the genotype obtained from this marker was treated as the “true” genotype for this probe. The proportions of the genotyping calls for probes that were supported by these other markers were then determined.

## Supporting Information

Table S1 Conservation of probe sequences in Mo17 whole genome shotgun sequence.
(0.04 MB DOC)

Table S2 Number of polymorphic probes per chromosome.
(0.04 MB DOC)
