# Supplementary material for: High-Resolution Genotyping via Whole Genome Hybridizations to Microarrays Containing Long Oligonucleotide Probes
Source: PLoS One. 2010 Dec 2;5(12):e14178. doi: 10.1371/journal.pone.0014178 (PMC2996289; doi:10.1371/journal.pone.0014178)
Supplement: Table S1 — Conservation of probe sequences in Mo17 whole genome shotgun sequence. (0.04 MB DOC) [file pone.0014178.s001.doc]

| Supplemental table 1. Conservation of probe sequences in Mo17 whole genome shotgun sequence. | | | | | |  |  |  |
| --- | --- | --- | --- | --- | --- | --- | --- | --- |
| CGH Marker Mo17 conservation | B>M (all^1^) | M>B (all^1^) | Total |  | B>M (2-FC^2^) | M>B (2-FC^2^) | Total |  |
| Perfect match (100% identity and coverage | 5,419 (3%) | 10,860 (52%) | 16,279 |  | 1,717 (1%) | 4,316 (51%) | 6,033 |  |
| Conserved (>90% identity and coverage) | 91,013 (44%) | 7,430 (35%) | 98,443 |  | 65,629 (40%) | 2,955 (35%) | 68,584 |  |
| No match (<90% coverage or identity) | 108,502 (53%) | 2,643 (13%) | 111,145 |  | 97,382 (59%) | 1,123 (13%) | 98,505 |  |
| Total | 204,934 | 20,933 | 225,867 |  | 164,728 | 8,394 | 173,122 |  |
|  |  |  |  |  |  |  |  |  |
| ^1^The B>M or M>B (all) refers to all probes with a FDR<0.05 in a comparison of B73 and Mo17. | | | | | | | | |
| ^2^The B>M or M>B (2-FC) probes refers to the subset of polymorphic probes that have a FDR <0.0001 and a minimum of 2-fold change between B73 and Mo17 | | | | | | | | |
