# Supplementary material for: High-Resolution Genotyping via Whole Genome Hybridizations to Microarrays Containing Long Oligonucleotide Probes
Source: PLoS One. 2010 Dec 2;5(12):e14178. doi: 10.1371/journal.pone.0014178 (PMC2996289; doi:10.1371/journal.pone.0014178)
Supplement: Table S2 — Number of polymorphic probes per chromosome. (0.04 MB DOC) [file pone.0014178.s002.doc]

| Supplemental table 2. Number of polymorphic probes per chromosome | | | | | |  |
| --- | --- | --- | --- | --- | --- | --- |
| Chromosome | B>M (all^1^) | B>M (2-FC^2^) | M>B (all^1^) | M>B (2-FC^2^) | B>M & M>B (2-FC^2^) combined | |
| 1 | 33419 | 26953 | 2931 | 1192 | 28145 | |
| 2 | 23256 | 18700 | 2496 | 961 | 19661 | |
| 3 | 23343 | 18944 | 2138 | 820 | 19764 | |
| 4 | 22397 | 17554 | 2634 | 1010 | 18564 | |
| 5 | 17957 | 14404 | 2076 | 850 | 15254 | |
| 6 | 20532 | 16805 | 1727 | 675 | 17480 | |
| 7 | 17625 | 14083 | 1886 | 770 | 14853 | |
| 8 | 15116 | 12108 | 1874 | 779 | 12887 | |
| 9 | 15324 | 12333 | 1559 | 644 | 12977 | |
| 10 | 15965 | 12844 | 1612 | 693 | 13537 | |
| Total | 204934 | 164728 | 20933 | 8394 | 173122 | |
|  |  |  |  |  |  | |
| ^1^The B>M or M>B (all) refers to all probes with a FDR<0.05 in a comparison of B73 and Mo17. | | | | | |  |
| ^2^The B>M or M>B (2-FC) probes refers to the subset of polymorphic probes that have a FDR <0.0001 and a minimum of 2-fold change between B73 and Mo17 | | | | | |  |
